# “What Good Is That?” – Perspectives of Low-Income Older Adults Using Telehealth for Serious Illness Conversation

**DOI:** 10.1093/geroni/igaf122.1721

**Published:** 2025-12-31

**Authors:** Kelseanne Breder, Christine Jacob, Daniel David

**Affiliations:** New York University, New York, New York, United States; Albany Medical College, Albany, New York, United States; New York University, New York, New York, United States

## Abstract

Telehealth offers an avenue for older adults to access providers for serious illness conversations. However, telehealth may be embraced by some and not by others. Urban-dwelling, low-income older adults have unique challenges in accessing continuous care, engaging with the healthcare system and receiving support to address serious illness care needs. To investigate telehealth acceptance, we conducted interviews with 46 residents of 3 Medicaid-funded assisted living facilities which provide social, functional, and clinical support to historically underserved populations. Interview transcripts were analyzed using conventional content analysis. We identified four qualitative themes. 1. Benefit: Telehealth Offers Convenience – Participants highlighted how telehealth facilitates access to healthcare appointments, making it easier to receive care. 2. Barrier: Technology Fluency and Access is Not Universal – Many described physical and technological barriers that limit their ability to use telehealth effectively. 3. Preference: General Distaste for Telehealth – This theme captures participants’ overall attitudes and gut reactions to telehealth, ranging from enthusiasm to skepticism. 4. Concern: Mistrust in Clinical Connection – Patients expressed concerns about trust, security, and the ability to build meaningful connections with providers through telehealth. A key finding is that telehealth, which is intended to expand access to care, may diminish trust in the therapeutic relationship between urban-dwelling low-income older adults and providers. We offer guidance to revise screening tools that assess urban-dwelling older adults’ comforts and readiness to engage with telehealth technology. We suggest amendments to the Department of Health and Human Services’ telehealth guidelines to improve equitable provision of telehealth services.

